# Prevalence and factors associated with overweight, obesity, and hypertension in a large clinical sample of adults with autism spectrum disorder

**DOI:** 10.1038/s41598-022-13365-0

**Published:** 2022-06-13

**Authors:** Robyn P. Thom, Michelle L. Palumbo, Christopher J. Keary, Jacob M. Hooker, Christopher J. McDougle, Caitlin T. Ravichandran

**Affiliations:** 1 Lurie Center for Autism, 1 Maguire Road, Lexington, MA 02421 USA; 2 Massachusetts General Hospital, 55 Fruit St, Boston, MA 02114 USA; 3 Department of Psychiatry, Harvard Medical School, 25 Shattuck St, Boston, MA 02115 USA; 4 Department of Pediatrics, Harvard Medical School, 25 Shattuck St, Boston, MA 02115 USA; 5 Department of Radiology, Harvard Medical School, 25 Shattuck St, Boston, MA 02115 USA; 6 McLean Hospital, 115 Mill Street, Belmont, MA 02478 USA

**Keywords:** Cardiology, Health care, Risk factors

## Abstract

Adults with autism spectrum disorder (ASD) are at risk for excess bodyweight and hypertension, yet the prevalence of and clinical predictors for these health conditions remain unknown. The objective of this study was to assess the prevalence of overweight, obesity, and hypertension in a large clinical sample of adults with a confirmed diagnosis of ASD and to examine potential clinical predictors. This retrospective chart review study included adult subjects (≥ 20 years) with ASD who had been seen within the past 5 years at a multidisciplinary developmental disorders clinic. Data collected from the electronic health record included age, sex, race and ethnicity, cognitive ability, language ability, body mass index (BMI), hypertension, and use of second generation antipsychotic medications (SGAs). Of 622 adults with a confirmed diagnosis of ASD potentially eligible for the study, 483 (78%) had one or more notes in their records from the past 5 years. Those with recent notes were 23% female, 89% White, and had a mean (SD) age of 28.1 (7.1) years. Overall prevalence estimates for adults represented by this predominantly male, White, and young clinical sample were 28% (95% CI 24%, 32%) for overweight (BMI 25–29.9 kg/m^2^), 35% (95% CI 31%, 40%) for obesity (≥ 30 kg/m^2^), and 11% (95% CI 9%, 15%) for hypertension. Controlling for age and sex, intellectual disability (ID) was significantly associated with BMI (p = 0.003) but not hypertension (p = 0.69); those with moderate or more severe ID had a mean BMI that was 2.26 kg/m^2^ (95% CI 0.96, 3.57) lower than those with no ID. Controlling for age and sex, neither language ability, Diagnostic and Statistical Manual of Mental Disorders, Fourth Edition (DSM-IV) subtype of autism, nor past or current use of SGAs were significantly associated with BMI or hypertension. The study identified a high prevalence of overweight and obesity in adults with ASD consistent with the prevalence of these medical comorbidities in the U.S. population.

## Introduction

Overweight and obesity, abnormal or excessive fat accumulation that may impair health, are major public health concerns. Excess bodyweight (overweight and obesity) is a significant and preventable risk factor for several other chronic diseases affecting virtually all major organ systems^1,2^. Obesity is associated with decreased life expectancy by 5–20 years^1^.

Adults with autism spectrum disorder (ASD) may be at increased risk for overweight and obesity for several reasons. First, excess bodyweight is known to be of significant concern in children with ASD. A recent meta-analysis demonstrated that children with ASD are 1.84 times more likely to experience obesity^3^ and childhood obesity is known to increase the risk of adult obesity by about five times in the general population^4^. Second, the core and associated symptoms of ASD leading to restricted food preferences, decreased physical activity, and use of food as a reward in some behavioral therapies, likely also contribute to excess bodyweight. Finally, the use of psychotropic medications, such as risperidone and aripiprazole, both second generation antipsychotic medications (SGAs) with approval by the Food and Drug Administration for the treatment of irritability in ASD, are known to be associated with weight gain^5^.

Adults with ASD are at as great or even greater risk for excess bodyweight and associated cardiometabolic risk factors than the general population. Three large medical registry based studies of adults^6^, transition-aged individuals (14–25 years)^7^, and older (ages ≥ 65 years) adults^8^ found increased rates of obesity for individuals with an ASD diagnosis. The study of older adults also found that those with ASD were twice as likely to have a diagnosis of hypertension^8^. While medical registry studies allow for the inclusion of large study populations, a common associated limitation is lack of diagnostic confirmation of ASD and other health diagnoses. In contrast to the medical registry studies, two smaller, retrospective studies of patients with clinically validated ASD diagnoses, one based on National Health and Nutrition Examination Survey (NHANES) data^9^ and one based on data from a single health system^10^, reported similar prevalence of obesity and hypertension for adults with and without ASD, with the exception of a slight increase in prevalence for adults 18–29 years for the NHANES based study^10^. Considering these mixed findings, studies assessing the prevalence of excess bodyweight and associated cardiometabolic risk factors utilizing larger populations with a clinically validated diagnosis of ASD in adults are needed. Furthermore, few studies have reported on whether any of the Diagnostic and Statistical Manual of Mental Disorders, Fourth Edition (DSM-IV)^11^ ASD subtypes (autistic disorder, Asperger’s disorder, or pervasive developmental disorder-not otherwise specified [PDD-NOS]) or other clinical features such as cognitive and language ability or psychotropic medication use are associated with these serious health conditions.

Better understanding the prevalence of overweight and obesity as well as their clinical predictors in adults with ASD is of critical clinical importance. Although much of ASD research is focused on the pediatric population, individuals with ASD spend most of their lives and receive the majority of their healthcare as adults^12^. Furthermore, because weight generally increases with age, while childhood obesity is a clear risk factor for obesity in adulthood, 70% of obese adults do not have a childhood history of obesity^4^. This is of clinical relevance because weight gain during adulthood is known to be associated with morbidity and mortality. Even modest weight gain after the age of 18 years increases the risk of coronary heart disease and type 2 diabetes mellitus irrespective of initial bodyweight^13^. Autism spectrum disorder is associated with reduced life expectancy and premature mortality^14–16^. The standardized mortality ratio for circulatory causes of death in a cohort of individuals with ASD from California compared to the general population is 2.3^15^. Similarly, the risk of death due to a circulatory cause was increased by 1.5 times in individuals with ASD in a Swedish population study^16^. While the cause of excess mortality remains unknown, the health conditions associated with excess bodyweight including cardiovascular disease, the leading cause of death in the United States (U.S.)^17^, are likely major contributors. Since people with mental disorders, and in particular those with intellectual disability (ID)^18,19^, receive less screening and lower quality treatment for cardiovascular diseases^20^, research focused on understanding and eventually reducing upstream risk factors for cardiovascular disease is needed.

This study aimed to assess the prevalence of overweight, obesity, and hypertension in a large clinical sample of adults with a confirmed ASD diagnosis, as well as to examine whether any clinical features associated with ASD including cognitive ability, language ability, DSM-IV ASD subtype, and the past or current use of SGAs are related to weight and hypertension.

## Methods

### Setting and sample

This retrospective chart review study was conducted at the Massachusetts General Hospital (MGH) Lurie Center for Autism. Individuals included were originally identified for a study investigating non-cardiometabolic medical comorbidities in adults with ASD using the Research Patient Data Registry (RPDR), a data warehouse collecting and storing historic and current clinical data on patients from the Mass General Brigham hospital system. Permission to access patient data is governed by the Mass General Brigham Institutional Review Boards. Adults screened for eligibility for the original study were ≥ 18 years old as of December 31, 2015 and had ≥ 3 occurrences of ASD-related keywords (“pervasive developmental disorder”, “Asperger syndrome”, “autistic disorder”, or “autism spectrum disorder”) in their electronic health records (EHR).

Electronic health records identified by keyword search were reviewed for diagnosis of ASD between 2016 and 2019. Eligibility criteria, confirmed using EHR review were: (1) past or current patient at the Lurie Center; (2) documented developmental history; (3) comprehensive clinical evaluation by a Lurie Center or MGH developmental pediatrician, psychiatrist, psychologist, neuropsychologist, or neurologist; and (4) support for an ASD diagnosis. Patients with a known genetic condition or chromosomal change of established association with ASD or ID were excluded. A minimum of two and maximum of three Lurie Center psychiatrists (C.J.M., M.L.P., C.J.K.) independently reviewed the most comprehensive clinical evaluation and supporting records and either confirmed an ASD diagnosis and made a DSM-IV subtype classification, or made a determination of other diagnosis, or insufficient information. Review by multiple psychiatrists was required to improve diagnostic accuracy.

Adults with an ASD diagnosis confirmed by ≥ 2 psychiatrists were included in the current study. The current study also required availability of follow-up patient data from the RPDR and ≥ 1 visit notes in the EHR from a primary care physician, psychiatrist, psychologist, or neurologist from the 5-year period beginning July 4, 2014. Both the original and the current study were approved by the local institutional review board (Mass General Brigham Institutional Review Board), with the current study approved as exempt. Informed consent was waived by the Mass General Brigham Institutional Review Board. All methods were carried out in accordance with relevant guidelines and regulations.

### Demographic and clinical characteristics

Age, sex, and race and ethnicity were obtained from the RPDR. All other data were collected retrospectively by manual chart review of the EHR using a cutoff date of July 3, 2019.

The presence and severity of ID were categorized based on the full scale intelligence quotient (FSIQ), if available, as mild, moderate, severe, or profound based on DSM-IV criteria. If FSIQ score was unavailable, presence and severity were categorized based on the DSM, Fifth Edition (DSM-5) criteria for ID based on level of adaptive functioning described in the medical record. Language ability was categorized as non-verbal, single words, phrase speech, simple sentences, or fluent speech.

A DSM-IV diagnosis of ASD was classified based on consensus of two or three Lurie Center psychiatrists. In the event that two or three psychiatrists confirmed an ASD diagnosis, but no two psychiatrists made the same DSM-IV subtype classification (autistic disorder, Asperger’s disorder, or PDD-NOS), the individual was included in the study, but DSM-IV subtype was considered missing.

### Cardiometabolic outcomes

For cardiometabolic outcomes, the 5-year follow-up period was defined as the 5 years prior to the cutoff date for EHR review, or, for individuals who died before July 3, 2019, the 5-year period prior to date of death.

Overweight and obesity were defined as a body mass index (BMI) of 25–29.9 kg/m^2^ and ≥ 30 kg/m^2^, respectively. Severe obesity was defined as a BMI ≥ 40 kg/m^2^. BMI was calculated based on the most recent height and weight in the EHR. If height and weight were not documented in the EHR, BMI was considered missing.

Hypertension was defined as a documented clinical diagnosis of hypertension with an accompanying treatment plan or three or more systolic blood pressure values ≥ 140 mmHg or three or more diastolic blood pressure values ≥ 90 mmHg documented in the EHR during the follow-up period. Hypertension was considered missing if there was no recorded diagnosis of hypertension and two or fewer blood pressure values documented within the follow-up period.

A history of tobacco use was also abstracted from the EHR. A history of diabetes and hyperlipidemia were collected but not reported due to poor data quality.

### Medication usage

Current SGA use was defined as an active prescription within 1 month of the chart review cutoff date. Past use was defined as a prescription that had been discontinued prior to the cutoff date.

### Statistical methods

Characteristics of adults with and without notes in their EHR from the follow-up period were compared using Fisher’s exact test for categorical variables and Welsh’s *t* test for age at EHR review. Prevalence of overweight, obesity, severe obesity, and hypertension were estimated for the full sample, by sex, by age and sex category, and by race and ethnicity category. Stratified estimates were reported for subgroups size ≥ 5. A post-hoc sensitivity analysis assessed impact of application of general BMI thresholds for overweight and obesity for Asian patients by recalculating overweight and obesity prevalence for Asian patients using alternate criteria of 23.0 kg/m^2^ ≤ BMI < 27.5 kg/m^2^ for overweight and BMI ≥ 27.5 kg/m^2^ for obesity.

Candidate clinical covariates were associated with BMI and hypertension using linear regression with robust standard errors for BMI and relative risk regression for hypertension. All models included age and sex as covariates and assumed a linear association with age in the absence of statistical evidence (p < 0.10) that including a covariate for squared age improved model fit. Candidate clinical predictors were entered one at a time into separate regression models for BMI and hypertension to estimate associations. Multivariable model building required two or more candidate clinical predictors with a p-value < 0.10 after adjustment for age and sex.

Individuals missing values for BMI and hypertension were excluded from corresponding prevalence estimation. For regression model fitting, missing data were imputed using multiple imputation with chained equations as implemented using Stata (version 14) statistical software, with 1000 imputations. Categories with < 10% frequency were combined into larger categories when appropriate or else excluded from analysis prior to imputation. Variables included in the chained equations were age (linear and quadratic terms), sex, ID, language ability, DSM-IV diagnosis of autistic disorder, SGA use, BMI, and hypertension.

Ninety-five percent confidence intervals for prevalence were calculated using Wilson’s method. Relative risk regression used the modified Poisson approach to prospective studies using binary data^21^. Statistical tests were two-sided and conducted at the test-wise alpha = 0.05 significance level. Statistical tests associated with regression models accounted for uncertainty in variance estimation due to imputation of missing data. Comparisons of demographic and clinical characteristics using the observed data and prevalence estimation were conducted using version 9.4 of SAS (SAS Institute, Cary, NC). Multiple imputation using chained equations and regression analyses were conducted using version 14 of Stata (StataCorp, College Station, TX).

## Results

### Study eligibility

**Figure 1:**
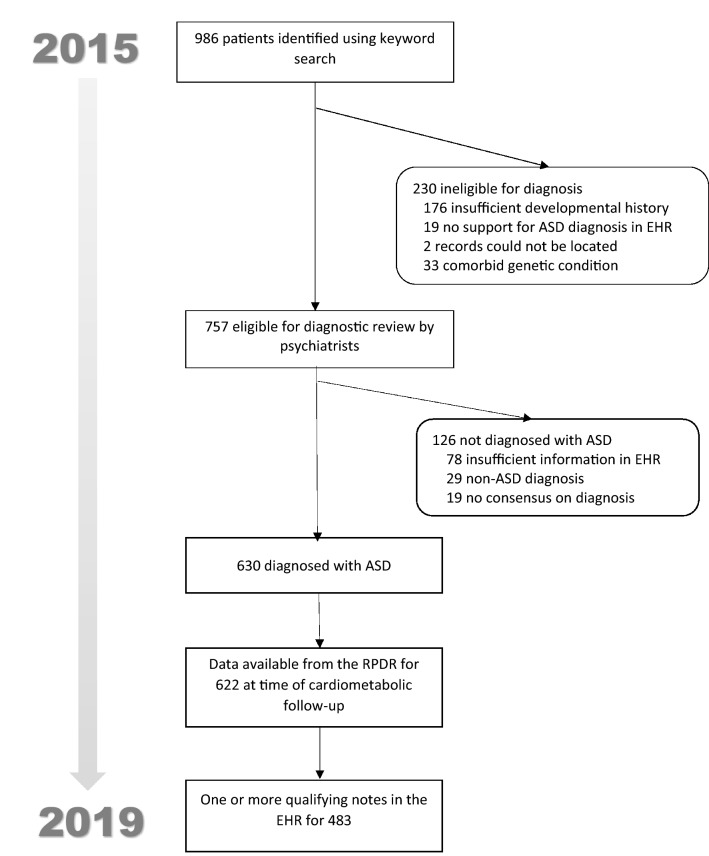
Number of patients meeting eligibility criteria for original study and cardiometabolic follow-up study. *ASD* autism spectrum disorder, *EHR* electronic health records, *RPDR* research patient data registry.

Flow of patients through the eligibility confirmation and diagnostic process is reported in Fig. 1. Of 622 adults with a confirmed diagnosis of ASD potentially eligible for the study, 483 (78%) had one or more notes in their EHR from the past 5 years. Adults with ≥ 1 notes were significantly more likely to reside in Massachusetts (p < 0.001, Fisher’s exact test). There were no other significant differences in demographic characteristics or DSM-IV subtype frequencies between those with and without notes (Table 1).

**Table 1:** Characteristics of ASD patients with and without one or more notes from the past 5 years in their electronic health record.

	Full sample n = 622	One or more notes n = 483	No notes n = 139	p^1^
Female^2^, n (%)	137 (22%)	109 (23%)	28 (20%)	0.64
Age (years), Mean (SD; Range)	28.1 (7.1; 20–65)	28.1 (6.9; 20–65)	28.4 (8.0; 21–61)	0.69
**Race and ethnicity, n (%)**^**3**^				0.45
Asian	25 (4%)	19 (4%)	6 (4%)	
Black or African American	26 (4%)	19 (4%)	7 (5%)	
Hispanic or Latino	14 (2%)	12 (3%)	2 (1%)	
White	528 (88%)	412 (89%)	116 (87%)	
Other	6 (1%)	3 (0.7%)	3 (2%)	
**Marital status, n (%)**^**3**^				0.21
Single	598 (97%)	463 (97%)	135 (98%)	
Married	3 (0.5%)	2 (0.4%)	1 (0.7%)	
Separated	1 (0.2%)	1 (0.2%)	0 (0%)	
Divorced	1 (0.2%)	0 (0%)	1 (0.7%)	
Other	14 (2%)	13 (3%)	1 (0.7%)	
**State of residence**^**3,4**^				< 0.001
Massachusetts	544 (88%)	448 (93%)	96 (70%)	
Other Northeastern United States	65 (10%)	29 (6%)	36 (26%)	
Other United States, Outside Northeast	12 (2%)	6 (1%)	6 (4%)	
**DSM-IV diagnosis, n (%)**^**3**^				0.87
Autistic disorder	428 (78%)	334 (77%)	94 (78%)	
Asperger’s disorder	65 (12%)	50 (12%)	15 (13%)	
PDD-NOS	58 (11%)	47 (11%)	11 (9%)	

*DSM-IV* Diagnostic and Statistical Manual of Mental Disorders, Fourth Edition, *PDD-NOS* pervasive developmental disorder-not otherwise specified.

^1^Fisher’s exact test and Welsh’s *t* test compared characteristics of patients with and without notes from the past 5 years in their electronic health record.

^2^All patients were identified as either male or female in the patient data registry.

^3^Race and ethnicity categories were taken from the patient data registry. Race was not reliably identified for Hispanic or Latino adults. Race was missing for 18 adults (4%) with one or more notes from the past 5 years and 5 adults (4%) without notes from the past 5 years. Marital status was missing for 4 adults (0.8%) with one or more notes and 1 adult (0.7%) without notes. State of residence was missing for 1 adult (0.7%) without notes. DSM-IV diagnosis was missing for 52 adults (11%) with one or more notes and 19 adults (14%) without notes.

^4^Other Northeastern states represented were New Hampshire (n = 31), Connecticut (n = 9), New York (n = 6), Vermont (n = 6), New Jersey (n = 4), Rhode Island (n = 4), Maine (n = 2), Pennsylvania (n = 2), and Maryland (n = 1). States outside the Northeastern United States represented were Florida (n = 6), Alabama (n = 1), Arizona (n = 1), California (n = 1), Michigan (n = 1), North Carolina (n = 1), and Wisconsin (n = 1).

### Demographic and clinical characteristics of adults with ASD included in the study

Table 2 presents data for adults with notes in their EHR from the past 5 years. Twenty-three percent of included adults were female and 89% were White. Fifty-nine percent had some degree of ID, 17% were nonverbal, and 77% with an assigned DSM-IV subtype had an autistic disorder diagnosis.

**Table 2:** Clinical characteristics of ASD patients with electronic health record data from the past 5 years, based on observed data (n = 483) and multiple imputations^1^.

	n % Missing	Observed data	Multiple imputations
**Intellectual disability, n (%)**	1 (0.2%)		
None		196 (41%)	41%
Mild		126 (26%)	26%
Moderate^2^		125 (26%)	33%
Severe^2^		32 (7%)	
Profound^2^		3 (0.6%)	
**Language ability, n (%)**	0 (0%)		
Nonverbal		83 (17%)	17%
Single words^2^		46 (10%)	27%
Phrases^2^		53 (11%)	
Simple sentences^2^		32 (7%)	
Fluent		269 (56%)	56%
**DSM-IV diagnosis, n (%)**	52 (11%)^3^		
Autistic disorder		334 (77%)	73%
Asperger’s disorder^2^		50 (12%)	27%
PDD-NOS^2^		47 (11%)	
**Second generation antipsychotic use, n (%)**	2 (0.4%)		
Current		118 (25%)	25%
Past		150 (31%)	31%
No history		213 (44%)	44%

*ASD* autism spectrum disorder, *DSM-IV* Diagnostic and Statistical Manual of Mental Disorders, Fourth Edition, *PDD-NOS* pervasive developmental disorder-not otherwise specified.

^1^One thousand imputations were generated using chained equations with sex, age (linear and squared terms), intellectual disability, language ability, DSM-IV diagnosis of autistic disorder, second generation antipsychotic use, hypertension, and most recent body mass index (BMI) measurement from the past 5 years as covariates.

^2^Categories were combined prior to multiple imputation. Intellectual disability (ID) and language ability categories were combined due to low frequencies. Full scale IQ scores were available in the electronic health record for 119 (61%) adults with no ID, 54 (43%) adults with mild ID, 30 (24%) adults with moderate ID, 2 (6%) adults with severe ID, and 0 (0%) adults with profound ID. Classification of ID for adults without documented full scale IQ scores was based on level of adaptive functioning as documented in the record. Diagnoses of Asperger’s disorder and PDD-NOS were combined due to the strong association of Asperger’s disorder with fluent language and absence of ID.

^3^DSM-IV diagnosis was missing due to lack of agreement among diagnostic reviewers. For 25 of 52 patients, there was sufficient agreement that the appropriate diagnosis was not autistic disorder.

### Prevalence of risk factors for cardiovascular disease

**Figure 2:**
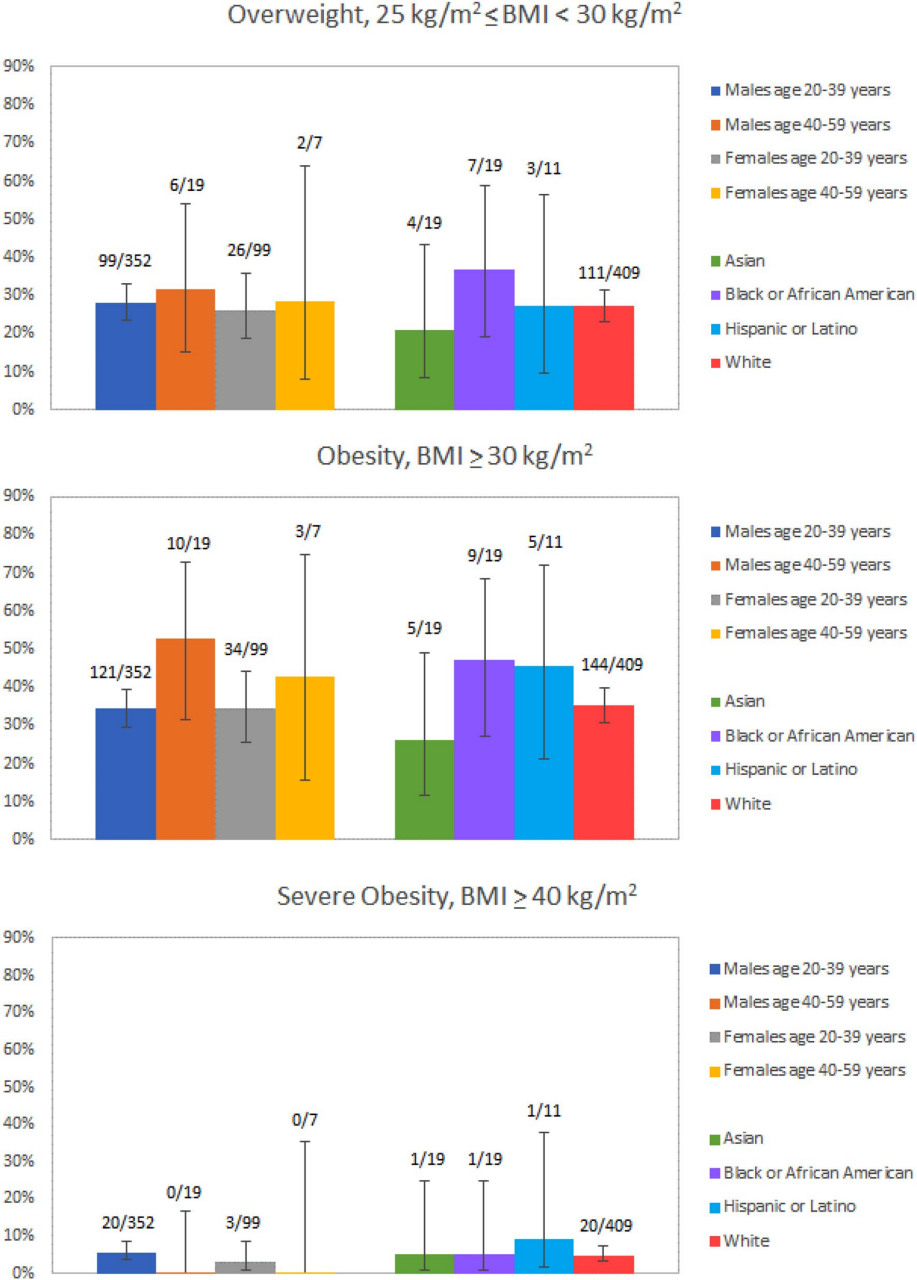
Sample prevalence of overweight, obesity (inclusive of severe obesity), and severe obesity in ASD patients by sex, age category (years), and race and ethnicity. Error bars correspond to the width of 95% confidence intervals for prevalence estimates. Observed frequencies are reported above error bars. Body mass index (BMI) data were missing for 5 adults: 2 males 20–39 years, 1 male 40–59 years, and 2 females 20–39 years; 3 White, 1 Hispanic or Latino, and 1 other race. Estimates are not reported for age 60 years and over because only one participant was over age 60 years. Race and ethnicity categories were taken from the patient data registry. Race and ethnicity was missing for 18 adults with BMI data.

**Figure 3:**
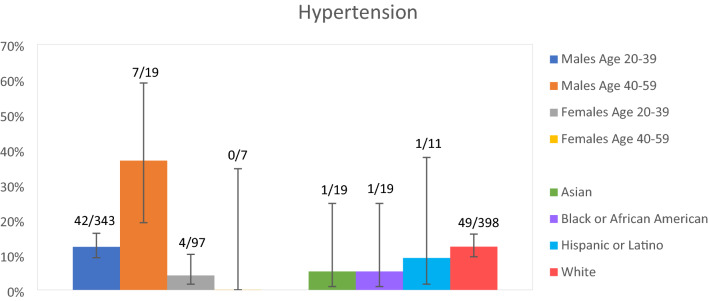
Sample prevalence of hypertension in ASD patients by sex, age category (years), and race and ethnicity. Error bars correspond to the width of 95% confidence intervals for prevalence estimates. Observed frequencies are reported above error bars. Data on hypertension were missing for 11 males age 20–39 years, 1 male age 40–59 years, and 4 females age 20–39 years. Estimates are not reported for age 60 years and over because only one participant was over age 60 years. Race and ethnicity categories were taken from the patient data registry. Race and ethnicity category was missing for 17 adults with hypertension data.

Across age groups and categories of race and ethnicity, prevalence of overweight was 133/478 (28%; 95% CI 24%, 32%) overall, 105/371 (28%; 95% CI 24%, 33%) for men, and 28/107 (26%; 95% CI 19%, 35%) for women. Prevalence of obesity was 168/478 (35%; 95% CI 31%, 40%) overall, 131/371 (35%; 95% CI 31%, 40%) for men, and 37/107 (35%; 95% CI 26%, 44%) for women; and prevalence of severe obesity was 23/478 (5%; 95% CI 3%, 7%) overall, 20/371 (5%; 95% CI 4%, 8%) for men, and 3/107 (3%; 95% CI 1%, 8%) for women. Figure 2 reports prevalence of overweight, obesity, and severe obesity by age and sex category and by race and ethnicity category, with age categorized according to U.S. National Center for Health Statistics (NCHS) data briefs^22,23^. Prevalence of hypertension was 53/467 (11%; 95% CI 9%, 15%) overall, 49/362 (14%; 95% CI 10%, 17%) for men, and 4/105 (4%; 95% CI 1%, 9%) for women. Figure 3 reports prevalence of hypertension by age and sex category and by race and ethnicity category. Though the NCHS reports hypertension for age 18–39 years, we report prevalence for 20–39 years since all adults in the study were at least 20 years old at EHR review. Nine of 479 adults (3%; 95% CI 1%, 4%) had any history of tobacco use. Using alternate thresholds for overweight and obesity for Asian adults increased estimated overweight prevalence to 37% (95% CI 19%, 59%) but did not change the prevalence estimate for obesity.

### Associations of demographic and clinical characteristics with metabolic risk factors

**Figure 4:**
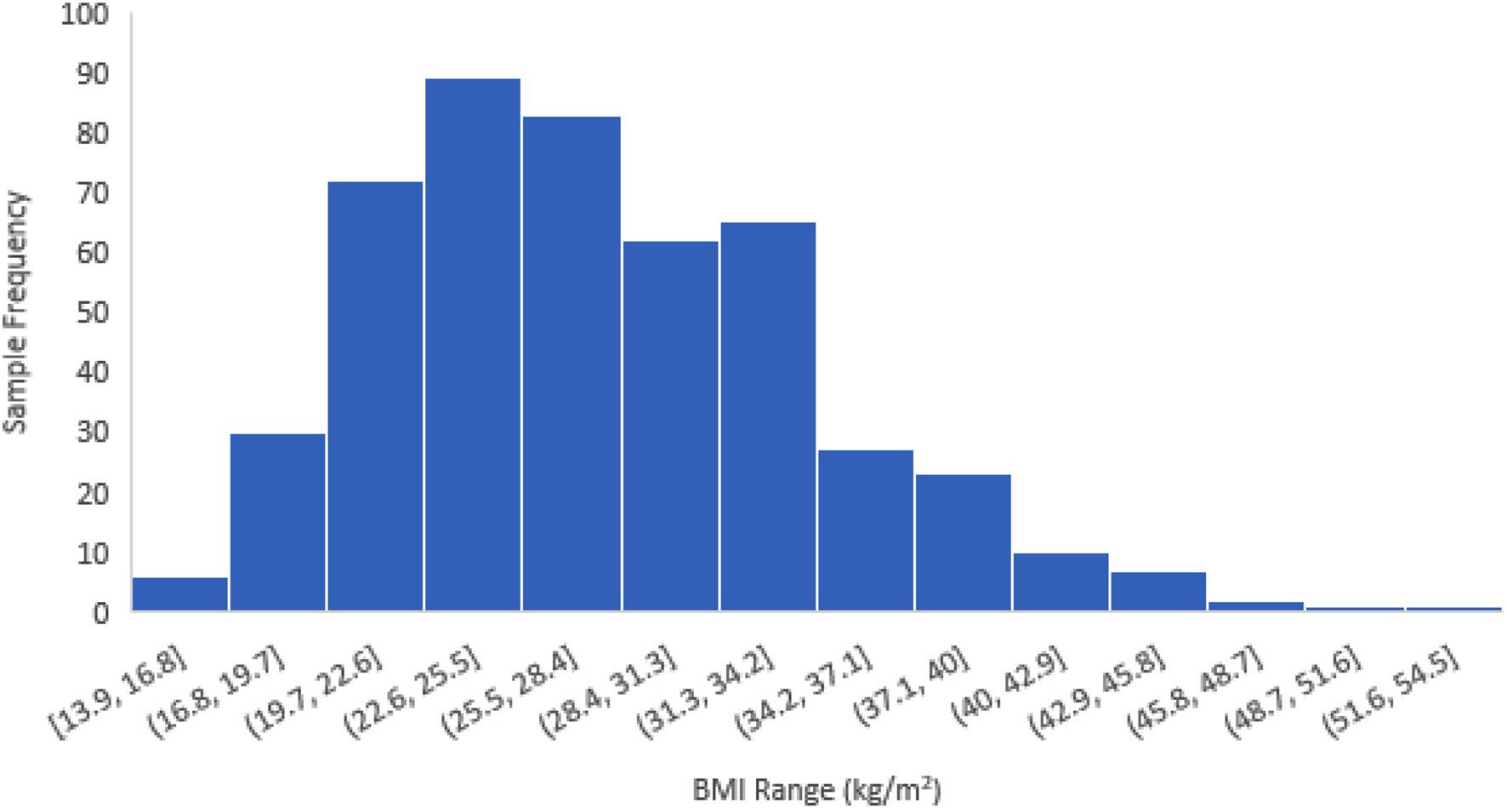
Histogram of most recent BMI measurement, n = 478. Five of the 483 adults in the sample did not have a body mass index (BMI) measurement from the past 5 years.

A histogram showing the sample distribution of most recent BMI measurement is provided in Fig. 4. The distribution is right-skewed, with a frequency of values in the underweight range (BMI < 18.5) of 3% (14/478, 95% CI 2%, 5%). Table 3 presents estimates for mean differences in BMI and relative risks for hypertension comparing patients with different demographic and clinical characteristics. Controlling for age and sex, ID was significantly associated with BMI (F_2,476_ = 5.99, p = 0.003); those with moderate or more severe ID had a mean BMI that was 2.26 kg/m^2^ (95% CI 0.96, 3.57) lower than those with no ID. No other clinical characteristics were significantly associated with BMI. Controlling for age and sex, neither ID, language ability, diagnosis of autistic disorder, nor past or current use of SGAs were significantly associated with hypertension. Male sex and older age were associated with higher risk of hypertension. Categorization of demographic and clinical characteristics for regression analysis and associated frequencies based on multiple imputation are shown in Table 2.

**Table 3:** Associations of potential demographic and clinical predictors with metabolic risk factors in 483 ASD patients.

	BMI		Hypertension	
	Mean difference (95% CI)	p	RR (95% CI)	p
Male	− 0.05 (− 1.42, 1.33)	0.95	3.7 (1.4, 10.1)	0.01
Age, 10 year increase	0.69 (− 0.20, 0.16)	0.13	1.6 (1.3, 1.9)	< 0.001
**Intellectual disability**		0.003		0.69
None (Ref)	–		–	
Mild	− 0.68 (− 2.17, 0.81)		1.2 (0.6, 2.2)	
Moderate or more severe	− 2.26 (− 3.56, − 0.95)		1.3 (0.7, 2.3)	
**Language**		0.12		0.27
Nonverbal (Ref)	–		–	
Some language	0.19 (− 1.69, 2.08)		0.7 (0.3, 1.3)	
Fluent	1.34 (− 0.40, 3.08)		0.6 (0.3, 1.1)	
**Autistic disorder**	− 0.45 (− 1.80, 0.90)	0.51	1.9 (0.9, 4.0)	0.10
**Second generation antipsychotic medication**		0.53		0.11
No history (Ref)	–		–	
Past history	0.72 (− 0.65, 2.09)		1.4 (0.7, 2.6)	
Current use	0.58 (− 0.88, 2.05)		1.9 (1.0, 3.4)	

*ASD* autism spectrum disorder, *BMI* body mass index. Estimates and 95% CIs are from risk regression (hypertension) and linear regression (BMI) models. All models included sex and age as covariates. Missing values were accommodated using multiple imputation for chained equations with sex, age (linear and squared terms), intellectual disability, language ability, DSM-IV diagnosis of autistic disorder, second generation antipsychotic use, hypertension, and most recent BMI measurement from the past 5 years as covariates.

## Discussion

This retrospective study evaluated the prevalence of overweight, obesity, and hypertension in a large clinical sample of adults with ASD. It also examined whether clinical features of ASD were associated with weight and hypertension. Sixty-four percent of males and 61% of females in the sample had BMI ≥ 25 kg/m^2^. Fourteen percent of males and 4% of females in the sample had hypertension.

The prevalence rates of overweight, obesity, and hypertension observed in this sample of adults with ASD are of clinical concern. First, the observed prevalence rates of overweight and obesity would be considered elevated according to recently proposed standardized prevalence thresholds^24^. The observed prevalence of BMI ≥ 25 kg/m^2^ (overweight or obese) of 64% for males and 61% for females falls within the “high” prevalence category. Furthermore, the observed prevalence of BMI ≥ 30 kg/m^2^ (obese) of 35% falls into “very high.” Notably, younger adults were overrepresented in our sample. Overrepresentation of younger adults likely underestimates the health burden of the several health conditions associated with obesity, including hypertension.

Taking into account the age and sex distribution of the sample, prevalence estimates for the study are consistent with prevalence estimates for the general United States population. For adults age 20–39 years, for example, 2017–2018 NCHS prevalence estimates for obesity were 40.3% for men and 39.7% for women^25^, which are slightly higher than the 34% estimates for both men and women age 20–39 years from this study. For adults age 18–39 years, 2015–2016 NCHS prevalence estimates for hypertension were 9.2% for men and 5.6% for women^23^, a difference of three or fewer percentage points from this study’s estimates of 12% for men and 4% for women in the 20–39 years age range. Ninety-five percent confidence intervals for NCHS estimates and estimates from this study overlapped for all estimates. However, it is important to note that prevalence rates of obesity vary by state and > 90% of our sample resided in Massachusetts, which has lower rates of obesity and hypertension than the U.S. as a whole. For example, according to Behavioral Risk Factor Surveillance System 2019 data, the U.S. overall prevalence of adult obesity was 31.4% (95% CI 31.1–31.6) which is higher than the Massachusetts prevalence rate of 25.2% (95% CI 23.9–26.5)^26^. Finally, the burden of managing comorbidities associated with obesity may be particularly challenging for adults with ASD due to patient-, provider-, and systems-levels factors that create barriers to accessing high quality medical care^27^.

The results from this study are largely consistent with previous literature. The observed prevalence of obesity (BMI ≥ 30 kg/m^2^) of 35% in this study is similar to the prevalence reported in two large medical registry studies from Kaiser Permanente Northern California^6,7^. In addition, similar to the results of this study, a case–control EHR analysis that compared adults with ASD and matched peers demonstrated similar likelihood of overweight, obesity, and hypertension^10^. However, other previous studies have reported higher rates of overweight and obesity in individuals with ASD than in the general population^6,8,28^. From 1999–2000 through 2017–2018, the age-adjusted prevalence of obesity in the U.S. has increased from 30.5 to 42.4%^22^. The increase in prevalence of obesity in the general population may obscure differences in obesity prevalence in subgroups of the population.

This was one of the first studies to investigate clinical factors associated with overweight, obesity, and hypertension in adults with ASD. Although it is probable that many of the same risk factors for overweight, obesity, and hypertension in the general population apply to those with ASD, mechanisms driving obesity in adults with ASD remain unknown. Whether the core and associated symptoms of ASD may render adults more susceptible to cardiometabolic risk factors is undetermined. Results from this study demonstrate that the DSM-IV diagnosis of autistic disorder was not associated with either BMI or hypertension, suggesting that high prevalence rates of overweight and obesity are observed across the full spectrum of ASD. Similarly, language ability was not found to be associated with either BMI or hypertension. Previously published pediatric studies did not identify an association between cognitive ability and weight in children with ASD^29,30^. In contrast to these pediatric findings, this study demonstrated that patients with at least moderate ID had a mean BMI that was 2.26 kg/m^2^ (95% CI 0.96, 3.57) lower than those without ID. This finding suggests that certain environmental factors associated with ID, such as supported living environments, may be protective against excess weight. We did not find expected associations between use of SGAs with BMI and hypertension. Of note, relative risk estimates for hypertension were 1.4 (95% CI 0.7, 2.6) for history of taking SGAs and 1.9 (95% CI 1.0, 3.4) for those currently taking SGAs. Therefore, our study cannot rule out modest to moderate associations of SGAs with hypertension. Neither past nor current use of SGAs was associated with BMI. These results are similar to the majority of pediatric studies indicating that use of psychotropic medications were not associated with BMI^29,31,32^, as well as a previously published prospective 25-year outcome study in adults^33^. Taken together, these results suggest that the use of SGAs is unlikely to be the main driver of excess bodyweight in adults with ASD. This finding highlights the need to identify the more significant contributors to excess weight in this population.

This study has several important limitations and must be interpreted in the context of our sample. First, since all patients had access to longitudinal care at a tertiary care multidisciplinary developmental disorders clinic and > 90% resided in Massachusetts, the prevalence estimates for hypertension and obesity from our sample may not generalize. Our sample was also predominantly younger adults. Another limitation of the study is the high percentage of White adults in the sample and reliance on information from the patient data registry for categorizing race and ethnicity. Additionally, while several classes of medications can impact weight, only one class of medication (SGAs) were included in the analyses. Another limitation was the use of FSIQ, when available, to categorize the presence and severity of ID, which does not take into account overall functioning. When the FSIQ was not available in the EMR, the presence and severity of ID was determined based on the level of adaptive functioning described in the EMR. This resulted in different approaches to classification ID. However, this approach to classification is consistent with clinical practice, where a neuropsychological evaluation is recommended when a patient’s level of cognitive functioning cannot be adequately assessed through clinical evaluation alone. Finally, the prevalence rates of obesity and hypertension were not compared to prevalence rates in matched local controls.

Our study also has several strengths. This is the largest sample of adults with independently verified ASD diagnoses and clinical anthropometric data to examine these cardiometabolic risk factors. This study was also the first to assess whether clinical features associated with ASD such as cognitive ability, language ability, DSM-IV ASD subtype, and use of SGAs were associated with BMI or hypertension in adults.

## Conclusion

The current study identified a high prevalence of overweight and obesity in adults with ASD consistent with prevalence in the U.S. population. Intellectual disability was negatively associated with BMI. Language ability, DSM-IV subtype of ASD, and SGA use were not associated with weight or hypertension.
